# Developing Social Enhancements for a Web-Based, Positive Emotion Intervention for Alzheimer Disease Caregivers: Qualitative Focus Group and Interview Study

**DOI:** 10.2196/50234

**Published:** 2024-04-25

**Authors:** Ian Kwok, Emily Gardiner Lattie, Dershung Yang, Amanda Summers, Paul Cotten, Caroline Alina Leong, Judith Tedlie Moskowitz

**Affiliations:** 1 Feinberg School of Medicine Northwestern University Chicago, IL United States; 2 BrightOutcome Buffalo Grove, IL United States; 3 University of California San Francisco San Francisco, CA United States

**Keywords:** Alzheimer disease, dementia, caregiving, eHealth, web-based interventions, positive emotion, stress, coping

## Abstract

**Background:**

Alzheimer disease is a degenerative neurological condition that requires long-term care. The cost of these responsibilities is often borne by informal caregivers, who experience an elevated risk of negative physical and psychological outcomes. Previously, we designed a positive emotion regulation intervention that was shown to improve well-being among dementia caregivers when delivered through one-on-one videoconferencing lessons with a trained facilitator. However, the format required significant resources in terms of logistics and facilitator time. To broaden the reach of the intervention, we aimed to develop the Social Augmentation of Self-Guided Electronic Delivery of the Life Enhancing Activities for Family Caregivers (SAGE LEAF) program, an iteration of the intervention in a self-guided, web-based format with enhanced opportunities for social connection.

**Objective:**

The aim of this study was to gather feedback to inform the design of social features for the SAGE LEAF intervention. In the absence of a facilitator, our goal with the self-guided SAGE LEAF intervention was to integrate various social features (eg, discussion board, automated support, and profiles) to maximize engagement among participants.

**Methods:**

Qualitative data were collected from 26 individuals through (1) interviews with participants who completed a previous version of the intervention via videoconferencing with a facilitator, (2) focus groups with dementia caregivers who had not previously experienced the intervention, and (3) focus groups with Alzheimer disease clinical care providers. We conducted a qualitative thematic analysis to identify which social features would be the most helpful and how they could be implemented in a way that would be best received by caregivers.

**Results:**

Interview and focus group feedback indicated that participants generally liked the potential features suggested, including the discussion boards, multimedia content, and informational support. They had valuable suggestions for optimal implementation. For example, participants liked the idea of a buddy system where they would be matched up with another caregiver for the duration of the study. However, they expressed concern about differing expectations among caregivers and the possibility of matched caregivers not getting along. Participants also expressed interest in giving caregivers access to a podcast on the skills, which would allow them to review additional content when they wished.

**Conclusions:**

Taken together, the discussions with caregivers and providers offered unique insights into the types of social features that may be integrated into the SAGE LEAF intervention, as well as implementation suggestions to improve the acceptability of the features among caregivers. These insights will allow us to design social features for the intervention that are optimally engaging and helpful for caregivers.

## Introduction

### Background

The impact of Alzheimer disease (AD) continues to broaden as the global average life expectancy grows [1,2]. Consequently, the number of individuals who will assume the role of a primary caregiver of a friend or family member with AD is expected to rise, with estimates indicating that informal care accounts for 40% of the total cost of care [3]. In the United States alone, this amounts to an estimated annual total of 18.6 billion hours of unpaid care [4].

At the individual level, the protracted nature of AD results in an extended caregiving role that intensifies as the care recipient’s health gradually declines [5]. For example, initial caregiving responsibilities may include assisting with activities of daily living such as providing transportation, preparing meals, and helping with chores [6]. However, in more advanced stages of the disease, caregivers often have to cope with agitation, aggression, and wandering [7] while shouldering an increasing logistical and financial burden of coordinating care [8,9].

The weight of these responsibilities comes at a cost to caregivers, who experience adverse mental health outcomes such as increased depression, anxiety, and suicidality [10-13] as well as negative consequences for physical health, demonstrated by increased sleep disturbance, fatigue, and undernutrition [14,15]. This, in turn, may lead to a decline in the quality of care and, subsequently, poorer outcomes for the care recipient [5].

In light of the growing recognition of the stress of AD caregiving, researchers are developing targeted interventions that offer a combination of psychoeducation, social support, and psychological support for caregivers [16,17]. Of note, researchers are increasingly using eHealth technologies that leverage electronic information and communication (eg, telehealth, mobile apps, and web-based applications) to broaden the dissemination of these resources [18,19]. After the onset of the COVID-19 pandemic, eHealth technologies found renewed significance when in-person AD support services were suspended, necessitating a rapid shift to telehealth offerings [20].

This study was the first step in the adaptation of an existing caregiver intervention into a socially enhanced web-based intervention—Social Augmentation of Self-Guided Electronic Delivery of the Life Enhancing Activities for Family Caregivers (SAGE LEAF). SAGE LEAF will comprise a positive emotion regulation curriculum that has been shown to be helpful for individuals experiencing significant life stress, including those with type 2 diabetes, metastatic breast cancer, HIV, and depression [21-24]. In a previous study, the intervention was also tailored specifically for dementia caregivers and delivered through videoconferencing by trained facilitators [25]. The intervention was effective at reducing symptoms of depression while improving self-reported physical health and positive emotion outcomes. However, the one-on-one facilitation that was provided for participants required a significant commitment of resources in terms of recruiting and training facilitators as well as an estimated 6 to 8 individual contact hours per participant over the course of 6 weeks. Hence, our goal was to tailor the self-guided, web-based version of the intervention for caregivers while incorporating unique *social features* that may help foster engagement among participants.

### Objectives

Specifically, our aim was to enhance *social presence*, defined by computer-human interaction researchers as the perception of others in a virtual environment [26-28]. The construct has been shown to be associated with enhanced perceived learning and satisfaction in e-learning environments [29]. However, to identify potential social features that may be helpful for AD caregivers, it is also necessary to first understand how they currently use social technologies to support their caregiving activities and emotional well-being. For example, caregivers may use discussion boards hosted by the Alzheimer’s Association [30] or on social platforms such as Facebook or Reddit [31]. Furthermore, in the context of the COVID-19 pandemic, caregivers are now increasingly reliant on these social technologies with the rapid shift from in-person to virtual support resources, which include videoconferencing support groups for caregivers [32].

Hence, to examine the preferences and requirements of AD caregivers, this study aimed to solicit feedback on a set of potential social features for the SAGE LEAF intervention. These were identified from a review that we conducted on social features that were being implemented on research-focused and commercial eHealth applications (I Kwok, unpublished data, May 2021) in consultation with study team members and developers who were involved in the design of previous versions of the intervention (Textbox 1).

We collected feedback through (1) individual interviews with caregivers who completed the previous version of the intervention [25], (2) focus groups with dementia caregivers who had not yet been exposed to the intervention, and (3) focus groups with clinical providers of people with AD. The findings will inform the types of social features to be included in the SAGE LEAF intervention and how they can be implemented in a way that is most beneficial for caregivers.

**List of social features.**

**Social feature and description**

- Peer groups or cohort: enrolling caregivers in groups so that they have a “cohort” of peers that progress together in the program. This allows group participation to occur synchronously in a “live” virtual setting or asynchronously in a manner that allows participants to engage with the intervention at their own pace in the context of an assigned cohort [33-35].
- Profiles: a profile page that may be shared with others. Examples of profile content include being able to choose an avatar and answering some questions about themselves. Such features involve varying levels of self-disclosure, data management, and personalization that may enhance the sense of the presence of others in the intervention [36-38].
- Private messaging: participants are able to send each other private messages. Some examples include messaging through SMS text messages [39,40] or commercially available applications such as Facebook and WhatsApp [41] or built into the eHealth intervention [42,43].
- Buddy system or matching: pairing participants who are going through the intervention at the same time. Some examples include matching participants who are going through the intervention at the same time or with someone who has previously completed the program as a “peer mentor” for each other or enrolling a partner whom the participant has an existing relationship with [44-46].
- Videoconferencing group: a facilitated group with participants through videoconference. Similar formats include support group and education or training videoconferencing sessions [47,48].
- Discussion board: a web-based discussion board on the content of the lessons. Possible discussion board enhancements include notifications for when other users like or comment on their posts [49-51].
- Automated support: this may include reminders or notifications for participants who are not logging in or feedback collected at the end of lessons. Such features have been shown to enhance adherence in eHealth interventions [52,53].
- Multimedia, videos, or podcasts: these forms of multimedia are commonly used to disseminate educational material in a way that engages participants, thereby promoting literacy and enhancing health-related outcomes [54]. This might include testimonials and quotes from previous participants or messages from the study team.
- Informational support: frequently asked questions or other information about how to connect with caregiver organizations, online support groups, and mental health care providers. Such resources may enhance a participant’s sense of perceived support and information competence [55].

## Methods

### Design

A combination of focus groups and interviews was conducted to solicit feedback on the short list of potential social features for the SAGE LEAF intervention. Subsequently, a qualitative analysis was conducted on the transcripts to identify and gather feedback on each feature.

### Sample and Sampling

Three groups of participants were recruited (Tables 1 and 2):

1. Individual interviews: dementia caregivers who participated in a previous version of the intervention [56]. We emailed individuals who had previously provided consent for recontact and provided them with information about the interviews. If they wished to participate, they completed a screener survey to determine whether they met the following eligibility criteria: (1) currently identifying as the primary family caregiver of a person with dementia, (2) ability to speak and read English, (3) access to high-speed internet, and (4) access to a webcam for videoconferencing. The interviews were conducted by the lead author (IK), who adhered to a semistructured interview protocol to guide the discussions. They lasted approximately 45 to 60 minutes, and topics included (1) the types of social connection technologies that participants use in their everyday life, (2) reactions to potential social features that may be implemented for SAGE LEAF (eg, private messaging, discussion board, and virtual profiles), and (3) solicitation of suggestions for other social features not previously mentioned.
2. Caregiver focus groups: dementia caregivers from Northwestern Memorial Hospital’s Cognitive Neurology and Alzheimer’s Disease Center (CNADC) were recruited for 2 focus groups comprising 5 caregivers each (n=10). We emailed caregivers who had previously provided consent to be contacted for research purposes through the CNADC and provided them with information about the focus groups. If they wished to participate, they were asked to complete a web-based screener survey to determine whether they met the following eligibility criteria: (1) currently identifying as the primary family caregiver of a person with dementia, (2) ability to speak and read English, (3) access to high-speed internet, and (4) access to a webcam for videoconferencing. The focus groups lasted approximately 90 to 120 minutes and were similar in content to the interviews.
3. Clinician focus groups: we contacted clinicians who provided care for people with AD or their family or informal caregivers (eg, physicians, nurses, and social workers) from the CNADC and the University of California, San Francisco’s Memory and Aging Center via email. Both are comprehensive research and care centers that treat AD; hence, clinicians are involved in a broad range of AD programs that integrate patient care, training, and research. Interested clinicians completed a screener survey where they could indicate their professional experience to determine whether they met the following eligibility criteria: (1) current employment as a clinician for patients with AD and their caregivers (eg, physicians, nurses, and social workers), (2) access to high-speed internet, and (3) access to a webcam for videoconferencing. One focus group was conducted for AD care providers (n=6).

**Table 1:** Caregiver participant characteristics (n=20).

Caregiver characteristics		Interview participants (n=10)	Focus group participants (n=10)	All caregivers (n=20)
**Gender, n (%)**				
	Female	8 (80)	7 (70)	15 (75)
	Male	2 (20)	3 (30)	5 (25)
**Race, n (%)**				
	Black or African American	0 (0)	1 (10)	1 (5)
	White	10 (100)	9 (90)	19 (95)
Age (y), mean (SD)		70.20 (9.48)	63.40 (6.52)	66.80 (8.65)
Years of caregiving, mean (SD)		7.50 (2.50)	4.80 (3.19)	6.15 (3.17)

**Table 2:** Provider participant characteristics (n=6).

Provider characteristics		Focus group participants
**Gender, n (%)**		
	Female	6 (100)
**Occupation, n (%)**		
	Nursing	3 (50)
	Social work	3 (50)
Years in practice, mean (SD)		17.67 (11.33)
Patients with AD^a^ (%), mean (SD)		77.67 (31.89)

^a^AD: Alzheimer disease.

### Data Extraction Procedure

We conducted a qualitative thematic analysis to identify (1) positive and negative feedback and implementation suggestions for each of the potential social features and (2) additional social features suggested by participants.

The interview and focus group recordings were transcribed and uploaded to the Dedoose qualitative and mixed methods research application (SocioCultural Research Consultants) in preparation for thematic analysis. Because the interviews and focus groups followed a similar structure, with questions asked about the same set of features, all transcripts were collated and analyzed together. In total, 2 independent coders performed the coding (IK and CL). First, they conducted open-ended coding of a test transcript to extract a preliminary list of codes. The coders met to discuss their observations, resolve differences in these observations, and refine the list of codes. An additional study team member (JM) was involved in reviewing the list to ensure that it captured the potential range of feedback. Next, to ensure a high level of agreement, the coders performed an interrater reliability test on 3 transcripts. While there is debate about the applicability of interrater reliability in interpretive qualitative research, its measurement allows for greater transparency and motivates adherence to the established coding guidelines [57]. During the test, a Cohen κ of 0.68 was achieved, which is regarded as a substantial level of agreement [58]. Subsequently, the coders met to resolve the discrepancies highlighted by the test and refined the codebook once again.

Next, the coders proceeded with a first round of independent coding that comprised 5 transcripts. They randomly selected one of the transcripts to be double coded and met to discuss any discrepancies in their coding. Finally, they proceeded with a second round of independent coding of the 5 remaining transcripts.

The codes were organized based on the type of social feature that they referenced. For *each* social feature, subcodes were created for positive feedback, negative feedback or barriers, and implementation suggestions. Additional codes were created for other social technologies mentioned (eg, WhatsApp and Facebook), web-based resources, and additional ideas suggested by participants. In the *Results* section, the results are categorized by social feature type. Quotes from participants are presented with the following identifiers: (1) “PX” to denote participants who completed a previous version of the intervention, (2) “CR” to denote caregivers who participated in the focus groups, and (3) “PR” to denote providers who participated in the focus groups. Due to the personal nature of the interviews and focus groups, the full transcripts are not publicly available.

### Ethical Considerations

The protocol for this study was approved by Northwestern University’s institutional review board (STU00215548) before conducting the study. Eligible participants were sent an electronic consent form via email delineating the risks and benefits of participation, at which point they could confirm their preference to participate or decline participation in the study. Data collected from REDCap (Research Electronic Data Capture; Vanderbilt University) surveys were stored on a secure, Health Insurance Portability and Accountability Act–compliant, and password-protected server at the university. The data were deidentified, with no identifying information linked to their feedback. Participants were paid US $25 for attending the virtual focus groups and interviews. Providers were not reimbursed for their participation.

## Results

### Peer Groups or Cohort

Participants generally liked the idea of being in a peer group, and caregivers drew comparisons to their previous participation in caregiving support groups. However, they expressed concern as to how they would be matched into groups:

I think if you want to have peers, you do need to match them up closely. By the progression of disease maybe.

CR2

I think that you have to be careful as to who’s in the peer group...I’ve been in other support groups where [caregivers were caring for] somebody had dementia, somebody with Alzheimer’s, and [another caregiver was caring for someone with] PPA (primary progressive aphasia). And they’ve got similarities, but they’re so drastically different.

CR3

Furthermore, participants had varying opinions on how caregivers should be grouped together. Some suggestions included grouping by age, relationship to the care recipient, or type of dementia. One participant also suggested that it may be helpful to group individuals based on their recreational interests instead of their caregiving status:

I think it’s also good to have in a peer group...And I don’t think gender or anything like that matters whatsoever, it’s more about where they are and what they’re doing at the time, with the same type of diagnosis.

CR1

Well, I think that general background is so important...You know, educational—the number of degrees you get isn’t as important as interests. Are you interested in art? Are you interested in gardening?...It’s one way that certainly people get together.

PX1

Hence, type of dementia or progression of the disease were the most preferred methods of grouping individuals. Participants in the focus groups and interviews were able to readily articulate their grouping preferences by tapping into their own experience of caregiving and their specific needs based on their care recipient’s diagnosis. This underscores the utility of segmenting future participants by type of dementia diagnosis.

### Profiles

There is a high degree of variability in how user profiles are used in eHealth platforms, ranging from toggling basic settings to more extensive social features such as managing invites, notifications, and social groups. Our proposed enhanced user profiles would allow participants to customize the way in which they present themselves in the virtual space and would help lay the groundwork for future social interaction on the SAGE LEAF platform. Such user profiles might include the ability to select an avatar or allow participants to display their name and other personal information if they choose to do so.

Similar to the feedback on the peer groups, focus group and interview participants were primarily interested in the caregiving-related characteristics of other users, such as the relationship between the caregiver and care recipient and type of dementia diagnosis:

Family relationship. If they’re the adult child or spouse/partner. I’ve also—if it’s the younger onset versus later onset.

PR1

I think people would be interested in knowing the diagnosis of the person the other caregivers are caring for. So, if someone is an adult child caring for their parent with Alzheimer’s, I feel like they would be interested in meeting other people in similar situations.

PR3

I *think age is a big factor. I’ve had a lot of people who are younger, like maybe family caregivers, who are more interested in talking with somebody their own age. Or around—near their own age.* [PR4]

Taken together, this feedback suggests that the information that is shared in the profiles may be similar to the variables by which participants might be grouped together. This emphasizes the synergy between these features and how they may foster a sense of shared identity among participants.

### Private Messaging

A private messaging function would allow participants to contact each other individually on their own and would function similarly to social networking platforms such as Facebook and Instagram that allow for direct messaging. Overall, feedback was positive, but one clinician articulated their concerns about privacy and security:

...in my support group there are people who request, you know, to be connected to each other. And I think I always try to make sure that I ask both parties before I connect them...So maybe the option to stay private or to be public.

PR5

Contrary to our expectations that caregivers may share similar concerns about security and privacy regarding being contacted, the feedback suggests that caregivers were open to the idea of being able to send and receive messages and expressed minimal hesitation toward the private messaging feature:

Oh, I think that would be fine. You know, I see nothing wrong with that. I think, you know, friendships could be formed out of that. And private support and like-minded thinking people...I see absolutely nothing wrong with that.

PX10

I think that’s fine. I think that would be good because you’re going to have to find a way to build trust, and then it’s also somebody who is in a similar circumstance than yourself. So, yeah, so that’s how I would look at it.

PX12

Hence, feedback was generally positive on the ability to connect with other caregivers through a private messaging feature. Participants emphasized that their potential willingness to use this feature was based on the assumption that other participants would be in a similar caregiving situation to theirs. For instance, participants indicated that the similarity of the diagnosis of their care recipient or whether they were also spousal or other family caregivers were important attributes that might influence their use of this feature.

### Buddy System or Matching

To maximize the sense of social presence that future participants might experience, we initially proposed a buddy system where participants would be paired up with a peer or “buddy”—either with someone who was going through the program at the same time as them or with participants who had previously completed the intervention. This social feature would complement the peer groups in that participants would be able to feel like they were part of a group while being able to connect individually with other participants. Alternatively, this could be deployed as a stand-alone feature in the event that there were not enough participants to form a cohort. Overall, we received mixed feedback from caregivers and clinicians on the concept of a buddy system.

In their feedback, caregivers expressed interest in this idea because it would provide accountability for progressing through the program to their potential buddies and enhance their motivation to engage with the content:

Oh, it totally would [be helpful]. Because I would be more concerned about disappointing the other person. “Oh, they need me! I have to check my email,” or “I have to check that text. I don’t want to disappoint them.”

CR3

I think it’s a great idea. You know, it would have been nice, if I had had one, but I was just flying by the skin of my teeth and sometimes I crash landed.

PX7

In contrast, clinicians expressed significant concerns about the implementation of a buddy system. They described past experiences with similar efforts where the matching was unsuccessful or burdensome and led to a disappointing experience for the caregivers involved:

I’ve tried connecting caregivers that I work with, and unless they really hit it off naturally in most cases it doesn’t work out.

PR5

I think there can be a problem in the two caregivers having really different expectations about what the relationship is going to be...I think it would add a level of burden to caregivers too...

PR2

Hence, while caregivers expressed enthusiasm for this idea, clinicians spoke from their own past experiences and were strongly against the idea of matching because they found it challenging to establish shared expectations and to anticipate whether caregivers who were matched would get along well. Therefore, it is unclear whether the benefits of the buddy system may be outweighed by potential complications that arise from these unanticipated social dynamics.

### Videoconferencing Group

At present, support groups form a crucial resource for AD caregivers, as demonstrated by the wide range of group programing in both virtual and in-person formats [17,59]. Hence, another possible feature was a videoconferencing group where participants would be able to log in at a given time during the week to connect with other caregivers with the specific focus on discussing the skills being taught in the program.

The onset of the COVID-19 pandemic hastened the transition of in-person support groups to web-based videoconferencing groups. This transition was demonstrated in the readiness that caregivers expressed in adopting these videoconferencing technologies. It should be noted that all the interviews and focus groups took place at the start of the pandemic:

I have been surprised that the Zoom meetings—I’ve gone to many of them...I should point out I’m 83 years old, okay?...via online, that sort of thing, would be very good for a person like me.

PX1

In terms of implementation suggestions, one participant highlighted that these videoconferencing groups would be a good addition to the program as long as participation was optional. This underscores the importance of building flexibility into the social features being offered as caregivers have competing demands or may simply prefer different features:

I think if you could make it as an offering but not a requirement...But I think you have to be understanding of the fact that not everybody’s going to be able to do that at the same time...it’s hard for me to commit weekly to a certain time.

PX10

One concern that was expressed by both caregivers and clinicians was the importance of making sure that the videoconferencing group discussions stayed on topic. Caregivers articulated various past experiences where their time was not spent efficiently because other participants deviated from the focus of the discussions:

...I would go and check out other groups, and that was always a real disappointment. And I would not go back to those when, you know, somebody would just insist on eating up the entire hour with their issues. And so that's a problem...

PX11

I would gravitate toward anything where there was some real-time moderation or facilitation, just to help keep the learning on track.

CR5a

The feedback suggests that videoconferencing groups can be helpful for caregivers. However, there was concern about the efficiency of these meetings, which could be addressed by having a facilitator who is able to moderate and guide the discussions. A facilitated group would allow participants to discuss the topics freely while ensuring that the time is directed toward the topics and skills taught in the program. Participants also liked the idea of having a portion of the videoconferencing group sessions be not necessarily related to caregiving or the positive emotion skills taught in the program, with several participants expressing interest in an informal happy hour where they could connect with each other casually.

### Discussion Group

Caregivers often seek information about their care recipient’s diagnosis, behaviors, and symptoms through the internet. Hence, many already participate in AD-specific discussion groups that are associated with the Alzheimer’s Association or informal groups that proliferate on social media platforms such as Facebook and Reddit. In line with our expectations, participants were generally open to the idea of using a discussion board. One clinician suggested that there may be some overlap with these existing platforms, which could present a barrier to participants using the discussion board:

...some of the feedback we get from caregivers is that, “You’re asking me to do something I already have a mechanism for doing that. So, I already have a way to share photos with people that I’m close to, it’s called Facebook or whatever. But you’re asking me to sort of do it in this different venue.” So that’s been a negative when you’re asking somebody to do something, that they already have a way to do that.

PR2

Furthermore, both participants and providers emphasized that the use of the discussion board would be contingent on how the benefits of engaging with it were conveyed to participants. Some of their suggestions included highlighting how the discussion board could amplify their practice of the skills or allow them to feel more connected with other participants in the study:

I think there are some advantages, and that if you really say the discussion board is to really talk about the skills or share examples of where you use the skills...And if you framed it so that—I could even see it as being a way to amplify the skills.

PR3

...to encourage people and say, “Hey, look at, you know, it’s normal for you to feel isolated and trying to get questions answered. It’s worth it to try and work with these tools.”

PX12

This feedback suggests that caregivers may be open to using the discussion boards, yet there were concerns about how these discussion boards might duplicate existing resources. Thus, it is essential to highlight the benefits of engaging with the discussion board associated with the positive emotion skills program to encourage its use. This may be in the form of prompts or reminders to participants about these benefits.

### Automated Support

As described in Textbox 1, automated support would comprise notifications or reminders that are sent out based on certain triggers, for example, if a participant does not log on to the platform for a certain number of days or if they endorse poor mood for an extended period. With automated support, the intention is to provide caregivers with a sense that their participation is valued and that we would be responsive to their level of engagement. Similar to the feedback collected in previous versions of the intervention, participants found the concept of reminders helpful but expressed the need for these messages to be framed in a way that was supportive and encouraging:

I guess that’s where I would give them points, and like, more like entice them rather than nag them.

PR5

Because when you first said it [automated support], it was totally irritating to me. I thought, “I’m doing this to take care of myself, and now you’re making me accountable?! I don’t have time today!” And then after you talked a little more, then I felt better about it...I think it’s how you frame it. Or how I frame it for myself.

CR3

You could try to be really empathetic and kind of understand why they didn’t get to it, versus the risk that if someone got an automated message that might just add to their sense of everything negative about why they haven’t done the skills.

PR2

Across the board, providers and caregivers reiterated the importance of supportive and encouraging messaging when implementing the automated support features. This underscores the importance of emphasizing the *rewarding* aspects of participation—instead of reprimanding or penalizing caregivers for not using the various features. Furthermore, it may be helpful for this supportive language to be integrated not only into the automated support features but throughout the intervention as well—for example, using the registration emails, videos, and podcasts as opportunities for cheerleading and supporting participants.

### Multimedia, Videos, or Podcasts

Multimedia content such as videos and podcasts may help enhance the perception that there are study staff members behind the program and other caregivers who are involved in the study. In previous versions of the intervention, caregivers worked one-on-one with a facilitator to learn the skills. To compensate for a lack of face time in this self-guided format, we proposed the addition of multimedia content to make the skill-building lessons more engaging by hearing directly from the team members involved in the development of the intervention. In their feedback, participants unanimously liked the idea of including this multimedia content:

I think that would be good. I mean, again, it takes the program out of being a program and puts it into a dialogue with someone. And I think it’s always good to see the face of the people who are running the program.

PX1

It might be encouraging for them to hear and see that they’re not alone, that others have gone through it and have come out on the other side.

PX14

Some participants suggested the idea of including a podcast as part of the program. This would allow caregivers to review the material at a time that is most convenient for them. This is consistent with feedback on other social features, in which participants suggested that flexibility may be helpful for caregivers who are busy:

And I like the idea of the podcast, so that you can do it on your time and when it’s convenient for you...ten, less than ten minutes here and there throughout the week...

CR1

I participate in a 30-day class right now...It is a five-minute podcast that she sends, along with a list of daily activities and a curriculum has been provided in advance. So, you know that the five-minute podcast is five minutes out of your day, and you can do that, it’s pretty easy to find five minutes.

CR4

The overwhelmingly positive feedback on the proposed multimedia social features demonstrates that participants are interested in the sharing of insights from the study team as well as from previous participants. In the absence of live communication, their feedback suggests that such multimedia features may be central to developing a sense of social presence.

### Informational Support

Caregivers often use web-based resources to seek information about providing care for their loved ones with dementia. While participants felt that informational support could be helpful, their feedback suggests that it was important for it to be targeted and provide specific information that was useful for caregivers:

But be real specific...the specific information is way more helpful.

PX6

...my husband’s diagnosis is not specifically Alzheimer’s...a lot of the things that have to do with the Alzheimer's Association don't apply to him...

PX10

...the referral...You know, a piece of paper with 20 different organizations on it were not helpful.

CR5a

Their feedback also suggests that many caregivers are discerning about such resources and sophisticated in their information search methods. Hence, the informational support provided by the intervention should be thorough and specific for it to be meaningful for participants. For example, participants indicated that it would be helpful if such resources were organized by geographic location or if they could be sorted in a way that would make it easy for participants to find the resources that are most helpful to them. Another approach would be to provide additional resources that relate specifically to the skills that are being taught.

### Other Social Features

We also collected feedback on other social features that might be helpful for caregivers. One participant suggested that the study team solicit participants’ input throughout the program to foster a sense of involvement. This has some similarities to the brief survey that we will provide at the end of each lesson asking participants to rate how they felt about the lesson on a scale from 1 to 5 stars. While this feature was not previously considered a social feature, the act of soliciting feedback provides participants with an opportunity to express their thoughts about the program and reinforces the sense that there is a study team who is collecting the feedback and trying to improve the intervention for the benefit of caregivers:

I think asking for opinions...getting involved in just what you’re doing and asking what I think. “Okay, what do you think of the program?” It’s certainly one way to get involved, as long as it’s done in such a way that it’s meaningful.

PX1

Participants also mentioned how the use of other platforms such as Instagram or Facebook may complement the intervention. The feedback suggests that creating a parallel dialogue on these already used platforms could foster an enhanced sense of social connection. One benefit to using these platforms is that participants would be able to connect with each other regarding the positive emotion skills across multiple platforms, which may enhance their learning. Participants also mentioned how the COVID-19 pandemic heightened their sense of social isolation; hence, the integration of these popular social networking platforms may help caregivers feel more connected as they complete the study:

I think if you had something that allowed people to respond to one another, whether it was a chat room...they create a Facebook group that is specific to that course...And that those people during that course can talk to each other, and every now and then the instructor chimes in if she feels that there’s something that she can add to it or some guidance. But I think something where people could connect would be nice.

PX10

I was thinking along the same lines of connecting on a specific theme, you mentioned gardening or cooking, I think those are the kinds of things that people do on Instagram or Facebook. But the WhatsApp group can be more private, so it can be formed just with the people who meet each other, and then they can share...

PR5

## Discussion

### Principal Findings

In this study, we collected feedback on social features that may be implemented for a web-based positive emotion skills intervention for AD and other dementia caregivers. Through (1) individual interviews with participants who completed a previous version of the intervention, (2) focus groups with dementia caregivers, and (3) focus groups with AD clinicians, we collected information about the specific needs and preferences of caregivers in the implementation of these social features. Participants provided a number of insights into how to implement these features in a way that may be best received by caregivers.

Overall, participants provided extensive feedback on the proposed features and how they could be best implemented. However, they had fewer suggestions for additional features that might enhance a sense of social connection. This may be because we asked participants open-ended questions about additional features toward the end of the interviews and focus groups, at which point they may have exhausted their ideas or there may have been overlap with our proposed features. Nonetheless, participants were engaged throughout the discussions and provided unique insights into how we could refine our feature set.

Participants generally liked the proposed social features and provided valuable suggestions for how they could be improved. One such example is the multimedia content proposed for the intervention, for which participants suggested a podcast format to allow caregivers to review this additional material at their convenience. This is similar to other exploratory eHealth interventions that have used this delivery format to enhance health literacy [60], weight loss [61], and self-compassion [62].

Other feedback related to the automated support features, in which participants emphasized the importance of providing encouragement to caregivers instead of shaming them for nonadherence. In a study of a physical activity intervention for older adults, it was found that, when the messaging was positively framed (ie, described in terms of the rewards and benefits of exercise as opposed to the costs of inactivity), participants’ pedometer readings indicated that they had walked more compared to those who received negative or neutral messaging [63]. Therefore, we could incorporate this positive framing, for example, if participants have not logged into the website for several days, and send personalized email messages letting them know that their participation is missed, while recognizing that caregivers have busy schedules, and reminding them that they might receive a boost in positive emotion by spending just a couple of minutes completing the home practice activities.

There were certain instances in which caregivers and clinicians differed in their feedback. For example, caregiver participants were generally open to the idea of being paired up with a buddy in the program. However, clinicians who had implemented similar programs were able to speak from their own experiences with attempts to match participants that were not successful based on differences in life experiences and expectations for engaging with a buddy program. Thus, although caregivers thought that they would enjoy a buddy feature, clinicians noted significant barriers to the implementation of this feature. Another example of disagreement between caregivers and clinicians was the private messaging feature, where one clinician highlighted concerns about privacy and security that caregivers did not report. Across eHealth interventions, researchers have far more information about how these platforms work and the accompanying benefits and risks compared to their participants [64]. Hence, researchers have an ethical responsibility to convey this information to participants. Recognizing these differing perspectives underscores the importance of integrating feedback from both caregivers and clinicians in refining these social features.

Participants were asked for additional suggestions for features that would enhance social connection or a sense of social presence. Their suggestions included soliciting feedback from caregivers as they progress through the program and using existing social media platforms to foster a sense of social connection beyond the SAGE LEAF intervention.

### Further Research and Implications

The feedback collected from the focus groups and interviews will be used to inform the development of the social features for the SAGE LEAF intervention. This will include developing a list of “trigger events” for the automated support features and wording the notifications or reminders in a way that would be supportive to participants. We will also include enhanced user profiles where participants can toggle how they would like to receive notifications and share more detailed information about their caregiving status to other users if they wish to. We will also include videos and podcasts where study team members will introduce each positive emotion skill and suggest methods for mastering it.

The feedback from the focus groups and interviews also helped clarify which social features may be potentially challenging to implement, such as the buddy system. In future versions of the intervention, a study team member could facilitate a matching process among participants. However, this may require additional resources to implement.

The feedback made clear that informational resources were extremely helpful for caregivers. However, it was apparent that caregivers already seek these resources through web-based groups or informational websites hosted by caregiver organizations. Furthermore, it appeared that this information is most helpful when it is specific and tailored for the caregiver and care recipient. Recognizing that the primary aim of the intervention was to deliver the positive emotion skills and not more general caregiving skills per se and acknowledging that it would take significant resources to successfully implement these informational support features, this feature is less likely to be prioritized for inclusion in future iterations of SAGE LEAF.

While this study focused on all the potential social feature enhancements (ie, discussion boards, podcasts, and automated notifications) intended for SAGE LEAF, future versions of the intervention could explore which enhancements are most effective by using a factorial design where participants are randomly assigned to different combinations of the features to determine which can be most helpful or may best enhance a sense of social connection. In a randomized controlled trial of a previous version of the intervention designed for individuals with depressive symptoms [50], we randomly assigned participants to different combinations of enhancements and found that facilitator contact in combination with virtual badges yielded the highest participant engagement. Given that SAGE LEAF will be entirely self-guided, future research should explore which features, individually and in combination, lead to the biggest impact on caregiver engagement and well-being. For example, user profiles may help caregivers disclose more information about themselves and their caregiving circumstances, which may then enhance the quality of the interactions that take place on the discussion boards. Additional research may also involve measuring the extent to which these combinations of features lead to measurable increases in *social presence*—which is hypothesized to mediate the relationship between the application of these social features and desired intervention outcomes.

### Strengths and Limitations

The feedback collected from caregivers and providers offered valuable perspectives not only on features that may be helpful and engaging but also on ways in which they may be implemented to best benefit caregivers. In addition, the combination of interview and focus group formats allowed for both individual feedback and group discussions to aid in the generation of ideas.

However, the semistructured format of the interviews and focus groups potentially limited the range of feedback collected. With our questions focusing primarily on the proposed features, this may have constrained the participants’ ability to provide novel ideas for new social features.

Another limitation to our study is the lack of ethnic diversity in our caregiver sample, which consisted of primarily White participants. To achieve a more diverse sample and perspectives in future studies, future research should oversample for underrepresented ethnic groups if needed.

### Conclusions

This study involved a qualitative analysis of focus groups and interviews with caregivers and clinicians to determine which social features might be most helpful in tailoring a self-guided positive emotion intervention for AD caregivers. The feedback collected suggests that the participants were mostly open and receptive to the innovative social features we proposed. However, their lived and professional experiences provided unique insights into how best to implement these features in a way that would be helpful and engaging for caregivers participating in future versions of SAGE LEAF.

## Abbreviations

AD: Alzheimer disease
CNADC: Cognitive Neurology and Alzheimer’s Disease Center
REDCap: Research Electronic Data Capture
SAGE LEAF: Social Augmentation of Self-Guided Electronic Delivery of the Life Enhancing Activities for Family Caregivers

## Data Availability

The data collected in the coding process may be obtained by contacting the first author (IK).

## References

[1] Lladó A, Froelich L, Khandker RK, Roset M, Black CM, Lara N, Chekani F, Ambegaonkar BM (2021). Assessing the progression of Alzheimer's disease in real-world settings in three European countries. J Alzheimers Dis.

[2] Maiese K (2018). Sirtuins: developing innovative treatments for aged-related memory loss and Alzheimer's disease. Curr Neurovasc Res.

[3] Prince MJ, Wimo A, Guerchet MM, Ali GC, Wu YT, Prina M (2015). World Alzheimer report 2015-the global impact of dementia: an analysis of prevalence, incidence, cost and trends. Alzheimer’s Disease International.

[4] Alzheimer's Association (2019). 2019 Alzheimer's disease facts and figures. Alzheimers Dement.

[5] Koca E, Taşkapilioğlu Ö, Bakar M (2017). Caregiver burden in different stages of Alzheimer's disease. Noro Psikiyatr Ars.

[6] Kokorelias KM, Gignac MA, Naglie G, Rittenberg N, MacKenzie J, D'Souza S, Cameron JI (2022). A grounded theory study to identify caregiving phases and support needs across the Alzheimer's disease trajectory. Disabil Rehabil.

[7] Gauthier S, Cummings J, Ballard C, Brodaty H, Grossberg G, Robert P, Lyketsos C (2010). Management of behavioral problems in Alzheimer's disease. Int Psychogeriatr.

[8] El-Hayek YH, Wiley RE, Khoury CP, Daya RP, Ballard C, Evans AR, Karran M, Molinuevo JL, Norton M, Atri A (2019). Tip of the iceberg: assessing the global socioeconomic costs of Alzheimer's disease and related dementias and strategic implications for stakeholders. J Alzheimers Dis.

[9] Deb A, Thornton JD, Sambamoorthi U, Innes K (2017). Direct and indirect cost of managing Alzheimer's disease and related dementias in the United States. Expert Rev Pharmacoecon Outcomes Res.

[10] Liu S, Liu J, Wang XD, Shi Z, Zhou Y, Li J, Yu T, Ji Y (2018). Caregiver burden, sleep quality, depression, and anxiety in dementia caregivers: a comparison of frontotemporal lobar degeneration, dementia with Lewy bodies, and Alzheimer's disease. Int Psychogeriatr.

[11] Coffman I, Resnick HE, Lathan CE (2017). Behavioral health characteristics of a technology-enabled sample of Alzheimer's caregivers with high caregiver burden. Mhealth.

[12] Puga F, Wang D, Pickering C (2020). Suicidal ideation in dementia family caregivers. Innov Aging.

[13] Ma M, Dorstyn D, Ward L, Prentice S (2018). Alzheimers' disease and caregiving: a meta-analytic review comparing the mental health of primary carers to controls. Aging Ment Health.

[14] Vicente de Sousa O, Mendes J, Amaral TF (2022). Association between nutritional and functional status indicators with caregivers' burden in Alzheimer's disease. Nutr Diet.

[15] Peng LM, Chiu YC, Liang J, Chang TH (2018). Risky wandering behaviors of persons with dementia predict family caregivers' health outcomes. Aging Ment Health.

[16] Cheng ST, Au A, Losada A, Thompson LW, Gallagher-Thompson D (2019). Psychological interventions for dementia caregivers: what we have achieved, what we have learned. Curr Psychiatry Rep.

[17] Piersol CV, Canton K, Connor SE, Giller I, Lipman S, Sager S (2017). Effectiveness of interventions for caregivers of people with Alzheimer's disease and related major neurocognitive disorders: a systematic review. Am J Occup Ther.

[18] Christie HL, Bartels SL, Boots LM, Tange HJ, Verhey FR, de Vugt ME (2018). A systematic review on the implementation of eHealth interventions for informal caregivers of people with dementia. Internet Interv.

[19] Sitges-Maciá E, Bonete-López B, Sánchez-Cabaco A, Oltra-Cucarella J (2021). Effects of e-health training and social support interventions for informal caregivers of people with dementia-a narrative review. Int J Environ Res Public Health.

[20] Ortelli P, Ferrazzoli D, Versace V, Saltuari L, Sebastianelli L (2021). The need for psychological, caregiver-centered intervention in the time of COVID-19. Alzheimers Dement (N Y).

[21] Cheung EO, Cohn MA, Dunn LB, Melisko ME, Morgan S, Penedo FJ, Salsman JM, Shumay DM, Moskowitz JT (2017). A randomized pilot trial of a positive affect skill intervention (lessons in linking affect and coping) for women with metastatic breast cancer. Psychooncology.

[22] Cohn MA, Pietrucha ME, Saslow LR, Hult JR, Moskowitz JT (2014). An online positive affect skills intervention reduces depression in adults with type 2 diabetes. J Posit Psychol.

[23] Addington EL, Cheung EO, Moskowitz JT (2020). Positive affect skills may improve pain management in people with HIV. J Health Psychol.

[24] Moskowitz JT, Carrico AW, Duncan LG, Cohn MA, Cheung EO, Batchelder A, Martinez L, Segawa E, Acree M, Folkman S (2017). Randomized controlled trial of a positive affect intervention for people newly diagnosed with HIV. J Consult Clin Psychol.

[25] Moskowitz JT, Cheung EO, Snowberg KE, Verstaen A, Merrilees J, Salsman JM, Dowling GA (2019). Randomized controlled trial of a facilitated online positive emotion regulation intervention for dementia caregivers. Health Psychol.

[26] Aragon SR (2003). Creating social presence in online environments. New Dir Adult Contin Educ.

[27] Lowenthal PR, Dasgupta S (2010). Social presence. Social Computing: Concepts, Methodologies, Tools, and Applications.

[28] Gunawardena CN, Zittle FJ (1997). Social presence as a predictor of satisfaction within a computer‐mediated conferencing environment. Am J Distance Educ.

[29] Richardson JC, Maeda Y, Lv J, Caskurlu S (2017). Social presence in relation to students' satisfaction and learning in the online environment: a meta-analysis. Comput Human Behav.

[30] Scharett E, Madathil KC, Lopes S, Rogers H, Agnisarman S, Narasimha S, Ashok A, Dye C (2017). An investigation of the information sought by caregivers of Alzheimer's patients on online peer support groups. Cyberpsychol Behav Soc Netw.

[31] Romero-Mas M, Gómez-Zúñiga B, Cox AM, Ramon-Aribau A (2020). Designing virtual communities of practice for informal caregivers of Alzheimer's patients: an integrative review. Health Informatics J.

[32] Brown EE, Kumar S, Rajji TK, Pollock BG, Mulsant BH (2020). Anticipating and mitigating the impact of the COVID-19 pandemic on Alzheimer's disease and related dementias. Am J Geriatr Psychiatry.

[33] Ronen K, Grant E, Copley C, Batista T, Guthrie BL (2020). Peer group focused eHealth strategies to promote HIV prevention, testing, and care engagement. Curr HIV/AIDS Rep.

[34] Currie CL, Larouche R, Voss ML, Trottier M, Spiwak R, Higa E, Scott DR, Tallow T (2022). Effectiveness of live health professional-led group eHealth interventions for adult mental health: systematic review of randomized controlled trials. J Med Internet Res.

[35] Liao J, Xiao HY, Li XQ, Sun SH, Liu SX, Yang YJ, Xu DR (2020). A social group-based information-motivation-behavior skill intervention to promote acceptability and adoption of wearable activity trackers among middle-aged and older adults: cluster randomized controlled trial. JMIR Mhealth Uhealth.

[36] Fisher CB, Bragard E, Bloom R (2020). Ethical considerations in HIV eHealth intervention research: implications for informational risk in recruitment, data maintenance, and consent procedures. Curr HIV/AIDS Rep.

[37] Standard E (2010). Human Factors (HF); personalization of eHealth systems by using eHealth user profiles (eHealth). European Telecommunications Standards Institute.

[38] Chen W, Quan-Haase A, Park YJ (2018). Privacy and data management: the user and producer perspectives. Am Behav Sci.

[39] Mulawa MI, LeGrand S, Hightow-Weidman LB (2018). eHealth to enhance treatment adherence among youth living with HIV. Curr HIV/AIDS Rep.

[40] Pouls BP, Vriezekolk JE, Bekker CL, Linn AJ, van Onzenoort HA, Vervloet M, van Dulmen S, van den Bemt BJF (2021). Effect of interactive eHealth interventions on improving medication adherence in adults with long-term medication: systematic review. J Med Internet Res.

[41] Simons LP, van den Heuvel WA, Jonker CM (2018). eHealth WhatsApp group for social support: preliminary results. Proceedings of the 31st Bled eConference Digital Transformation: Meeting the Challenges.

[42] Tiong SS, Koh ES, Delaney G, Lau A, Adams D, Bell V, Sapkota P, Harris T, Girgis A, Przezdziecki A, Lonergan D, Coiera E (2016). An e-health strategy to facilitate care of breast cancer survivors: a pilot study. Asia Pac J Clin Oncol.

[43] Ryan K, Dockray S, Linehan C (2019). A systematic review of tailored eHealth interventions for weight loss. Digit Health.

[44] Takeda H, Takatori K (2022). Effect of buddy-style intervention on exercise adherence in community-dwelling disabled older adults: a pilot randomized controlled trial. Clin Rehabil.

[45] van Buul AR, Derksen C, Hoedemaker O, van Dijk O, Chavannes NH, Kasteleyn MJ (2021). eHealth program to reduce hospitalizations due to acute exacerbation of chronic obstructive pulmonary disease: retrospective study. JMIR Form Res.

[46] Naidoo SS, Gathiram P, Schlebusch L (2015). Effectiveness of a Buddy intervention support programme for suicidal behaviour in a primary care setting. S Afr Fam Pract.

[47] Banbury A, Nancarrow S, Dart J, Gray L, Parkinson L (2018). Telehealth interventions delivering home-based support group videoconferencing: systematic review. J Med Internet Res.

[48] Marhefka S, Lockhart E, Turner D (2020). Achieve research continuity during social distancing by rapidly implementing individual and group videoconferencing with participants: key considerations, best practices, and protocols. AIDS Behav.

[49] Addington EL, Cheung EO, Bassett SM, Kwok I, Schuette SA, Shiu E, Yang D, Cohn MA, Leykin Y, Saslow LR, Moskowitz JT (2019). The MARIGOLD study: feasibility and enhancement of an online intervention to improve emotion regulation in people with elevated depressive symptoms. J Affect Disord.

[50] Moskowitz JT, Addington EL, Shiu E, Bassett SM, Schuette S, Kwok I, Freedman ME, Leykin Y, Saslow LR, Cohn MA, Cheung EO (2021). Facilitator contact, discussion boards, and virtual badges as adherence enhancements to a web-based, self-guided, positive psychological intervention for depression: randomized controlled trial. J Med Internet Res.

[51] Cheung EO, Addington EL, Bassett SM, Schuette SA, Shiu EW, Cohn MA, Leykin Y, Saslow LR, Moskowitz JT (2018). A self-paced, web-based, positive emotion skills intervention for reducing symptoms of depression: protocol for development and pilot testing of MARIGOLD. JMIR Res Protoc.

[52] Lentferink AJ, Oldenhuis HK, de Groot M, Polstra L, Velthuijsen H, van Gemert-Pijnen JE (2017). Key components in eHealth interventions combining self-tracking and persuasive eCoaching to promote a healthier lifestyle: a scoping review. J Med Internet Res.

[53] Mohr DC, Cuijpers P, Lehman K (2011). Supportive accountability: a model for providing human support to enhance adherence to eHealth interventions. J Med Internet Res.

[54] Aida A, Svensson T, Svensson AK, Chung UI, Yamauchi T (2020). eHealth delivery of educational content using selected visual methods to improve health literacy on lifestyle-related diseases: literature review. JMIR Mhealth Uhealth.

[55] Slev VN, Mistiaen P, Pasman HR, Verdonck-de Leeuw IM, van Uden-Kraan CF, Francke AL (2016). Effects of eHealth for patients and informal caregivers confronted with cancer: a meta-review. Int J Med Inform.

[56] Dowling GA, Merrilees J, Mastick J, Chang VY, Hubbard E, Moskowitz JT (2014). Life enhancing activities for family caregivers of people with frontotemporal dementia. Alzheimer Dis Assoc Disord.

[57] O’Connor C, Joffe H (2020). Intercoder reliability in qualitative research: debates and practical guidelines. Int J Qual Methods.

[58] Sim J, Wright CC (2005). The kappa statistic in reliability studies: use, interpretation, and sample size requirements. Phys Ther.

[59] Wennberg A, Dye C, Streetman-Loy B, Pham H (2015). Alzheimer's patient familial caregivers: a review of burden and interventions. Health Social Work.

[60] Semakula D, Nsangi A, Oxman M, Austvoll-Dahlgren A, Rosenbaum S, Kaseje M, Nyirazinyoye L, Fretheim A, Chalmers I, Oxman AD, Sewankambo NK (2017). Can an educational podcast improve the ability of parents of primary school children to assess the reliability of claims made about the benefits and harms of treatments: study protocol for a randomised controlled trial. Trials.

[61] Turner-McGrievy GM, Wilcox S, Boutté A, Hutto BE, Singletary C, Muth ER, Hoover AW (2017). The dietary intervention to enhance tracking with mobile devices (DIET mobile) study: a 6-month randomized weight loss trial. Obesity (Silver Spring).

[62] Biber DD, Rice K, Ellis R (2021). Self-compassion training within a workplace physical activity program: a pilot study. Work.

[63] Notthoff N, Carstensen LL (2014). Positive messaging promotes walking in older adults. Psychol Aging.

[64] Skär L, Söderberg S (2018). The importance of ethical aspects when implementing eHealth services in healthcare: a discussion paper. J Adv Nurs.

